# One Step Further: Integrating Evidence-Based Guidelines into Practice to Address Environmental Challenges at the Men’s 2026 FIFA World Cup

**DOI:** 10.1007/s40279-026-02415-6

**Published:** 2026-03-23

**Authors:** Chris J. Esh, Sarah Carter, Valérie Bougault, Olivier Girard, Dina C. Janse van Rensburg, Bryna C. R. Chrismas, Tim Meyer, Lee Taylor

**Affiliations:** 1 Aspetar, Orthopaedic and Sports Medicine Hospital, Doha, Qatar; 2 School of Sport, Exercise and Health Sciences, National Centre for Sport and Exercise Medicine (NCSEM), Loughborough University, Epinal Way, Loughborough, LE11 3TU UK; 3 Faculty of Health, Exercise and Sports Science, Sport, Performance, and Nutrition Research Group, School of Allied Health, Human Services and Sport, La Trobe University, Melbourne, Australia; 4 LAMHESS, Université Côte d’Azur, Nice, France; 5 School of Kinesiology, University of British Columbia, Vancouver, BC Canada; 6 School of Human Sciences (Exercise and Sport Science), The University of Western Australia, Perth, WA Australia; 7 Section Sports Medicine & SEMLI, Faculty of Health Sciences, University of Pretoria, Pretoria, South Africa; 8 Medical Advisory Panel, World Netball, Manchester, UK; 9 Institute of Sport and Preventive Medicine, Saarland University, Saarbrücken, Germany; 10 School of Sport, Exercise and Rehabilitation, Faculty of Health, University of Technology Sydney (UTS), Sydney, Australia; 11 Human Performance Research Centre, University of Technology Sydney (UTS), Sydney, Australia

## Abstract

The Men’s 2026 Fédération Internationale de Football Association (FIFA) World Cup (Canada, Mexico and the USA) will expose teams to an unprecedented combination of environmental challenges (heat, altitude, air pollution, allergens and travel). Conditions will vary significantly between host cities, creating a significant threat to player health and performance. These unique stressors may require teams to adopt and adapt strategies to best protect player health and performance. A related open access *Sports Medicine* review outlines these environmental challenges and the evidence-based guidelines to prepare for and mitigate their effects. Motivation for the present review stems from the unique practical and logistical complexities of ‘tournament football’—especially at the expanded 2026 FIFA World Cup—alongside the limitations of previous football-facing reviews, where ‘evidence-informed practice’ is often not underpinned by football-specific research and thus limits external/ecological validity. Building on the recommendations of the main review, this partner review focuses on integrating those guidelines into practice. It aims to provide teams with a practice-compatible framework to implement evidence-based strategies to protect player health and optimise performance at the 2026 FIFA World Cup.

## Key Points

**Table:** 

National teams participating in the Men’s 2026 Fédération Internationale de Football Association (FIFA) World Cup would benefit from adopting targeted strategies to prepare for and mitigate environmental challenges, including heat, altitude, air pollution, allergens and travel, to best protect player health and optimise performance throughout the tournament.
Evidence-based guidelines addressing heat, altitude, air pollution, allergens and travel are well-established; however, they are rarely underpinned by football-specific research, limiting their external/ecological validity, while practical and logistical barriers further hinder their adoption.
This article provides a practical framework for teams at the 2026 FIFA World Cup to integrate evidence-based guidelines with minimal disruption to football-specific preparations to best protect player health and optimise performance.

## Introduction

The Men’s 2026 Fédération Internationale de Football Association (FIFA) World Cup (FWC) will pose an unprecedented combination of environmental challenges that teams will benefit from mitigating against to protect player health and optimise performance. These challenges (heat, altitude, air pollution, allergens and travel) and mitigation strategies have been extensively outlined in a related review (herein referred to as the ‘main review’) [1]. We recommend that the main review [1] be read before reading this partner review. A brief scene setting summary of these FWC-specific environmental challenges is provided below.

### Heat

Extreme heat is forecasted in most host cities during the 2026 FWC, with 14 of the 16 venues typically experiencing wet bulb globe temperatures (WBGTs) of ≥ 28 °C [2], and six could see WBGTs between 30 and 35 °C [3] during the months of June and July. For reference, FIFA’s heat policy typically mandates 3-min hydration/cooling breaks at 30 and 75 min of play if the WBGT is ≥ 32 °C [4], but FIFA announced (7th December 2025 [FIFA. Players to benefit from hydration breaks at FIFA World Cup 2026™. 2025 [cited 2025 7th December]; Available from: https://inside.fifa.com/organisation/news/hydration-breaks-world-cup-2026-player-welfare.]) that all matches regardless of environmental conditions will have 3-minute cooling breaks at the 2026 FWC. The American College of Sports Medicine (ACSM) is more conservative, recommending event cancellation if the WBGT is ≥ 29 °C for non–heat-acclimatised individuals or ≥ 32.3 °C for heat-acclimatised individuals [5], given the severe risk of exertional heat-related illnesses (EHIs) and the potentially fatal exertional heat stroke (EHS) [6]. Beyond health risks, these conditions have detrimental impacts on physical [7, 8], cognitive [9–11] and technical/tactical performance [12–16].

### Altitude

Nine matches in Mexico will take place at moderate altitudes across two venues [Guadalajara: 1566 m (~ 133 mmHg—atmospheric *p*O_2_); Mexico City: 2240 m (~ 121 mmHg)]. At these elevations, aerobic capacity is impaired and recovery from high-intensity efforts is slower [17–19] in non-acclimatised individuals. Although technical skills are not typically impacted, physical performance decrements include reductions in total distance covered (~ 3 to 9%) and high-velocity running (21%) [20], which may impair tactical performance or necessitate tactical changes [17].

### Air Pollution and Allergens

The distribution of host countries and cities will expose players to a range of air pollutants and seasonal allergens. High levels of ozone (O_3_) are to be expected in Los Angeles (LA) and Mexico, and particulate matter < 2.5 µm (PM2.5) is likely to be a predominant pollutant in other cities [1]. Airborne pollen will still be present in most host cities [21–23]. Deteriorations in air quality and the interaction with air pollutants and extreme heat can have severe health consequences for sensitive individuals [24–26] and impact players’ physical and technical performance [27, 28]. The west coast host cities in the USA and Canada (LA, San Francisco, Seattle and Vancouver) are high risk sites for wildfires, which can drastically reduce forecasted air quality [29] across the USA [30] and may even necessitate match postponement, cancellation or relocation [31].

### Travel, Jet-Lag and Travel Fatigue

Arriving at the 2026 FWC will require long-haul travel for some teams, and crossing three or more time zones can result in jet-lag symptoms that may increase in severity as more time zones are crossed. Jet-lag is characterised by circadian misalignment, sleep disruption and increased risk of injury and illness [32–35]. Additionally, with frequent in-tournament travel up to three time zones and flight times of up to 7 h, players are likely to experience accumulated travel fatigue. Travel fatigue presents with symptoms such as sleep deprivation, disorientation and headaches [36, 37]. These travel demands and jet-lag/travel fatigue symptomatology can negatively impact player health and performance [32, 38].

Together, these environmental factors create a complex and unprecedented set of stressors that teams will need to address to protect player health and optimise performance throughout the tournament.

### Scope and Purpose of the Partner Review

This partner review builds on the recommendations of the main review [1], focusing on how evidence-based guidelines can be integrated into practice, to best protect player health and optimise performance from environmental challenges at the 2026 FWC. Previous football-facing reviews have often lacked ‘evidence-informed practice’ and are not underpinned by football-specific research, limiting external/ecological validity [1]. Tournament football presents distinct logistical barriers that can restrict the adoption of interventions, even when their effectiveness is well established. Indeed, football-specific training and preparation will likely be the priority when teams arrive at the 2026 FWC. They may not be willing and/or able to adopt environmental-focused interventions that disrupt training schedules [39] and/or add physical, physiological and/or psychological stress on the players [40], despite their high efficacy to protect health and performance in light of the environmental challenges. Furthermore, the real-world challenges of elite football, tournament football and individual circumstances may prevent some players from any given squad adopting the evidence-based guidelines presented herein, holistically and/or in part.

For example, domestic European leagues (where ~ 70% of players competed prior to the 2022 FWC [41]) will finish between 16 and 24 May 2026, giving players 16–30 days (depending on the date of the first world cup matches that take place from 11 to 17 June 2026) with their national teams squads before the first match of the 2026 FWC. However, players who participate in the Union of European Football Associations (UEFA) Champions League final on 30 May 2026 in Budapest may have as little as 9 days (up to 15 days) before their first match and therefore, may be required to travel separately to the Team Base Camp and arrive considerably later than the rest of the squad. South American and/or Asian players in Europe would have to undertake extensive travel commitments to join the squad in their home countries, which may place considerable strain upon players’ well-being—although, players may benefit from time spent in their home countries with family members, etc. Individual factors (e.g. high sensitivity to allergens/pollution, injury/illness) can be considered, and in such cases, more individualised practice may be appropriate. However, this may not always be feasible where a national and domestic team do not work in synergy, or when existing club commitments (fixtures, training, load management, etc.) preclude adoption of best practice [e.g. heat acclimation/acclimatisation (HA) exposures cannot begin]. Resource discrepancies between teams, rather than practice compatibility, may prohibit the use of one or more proposed mitigation strategies during preparation for, or competition at, the 2026 FWC. Here, it is suggested that the potential performance and/or health decrement/challenge from the anticipated environmental challenges are first considered. Specific challenges (e.g. high environmental temperatures) can then be prioritised and available mitigation strategies selected from a resource perspective (e.g. ice towels and ice slushies [42, 43] represent relatively low-cost, evidence-backed alternatives compared with costly squad-wide phase-change ice vest use [44, 45]). Finally, it must be acknowledged that while the strategies outlined in this article are evidence based and highly effective, they may not align well with team practices and/or culture. It would be naïve to expect all squads and players to want and/or be able to adopt all the presented recommendations ubiquitously before and during the 2026 FWC, given the challenges to practice and integration alluded to. In reality, each squad/player will adopt individualised (player- or squad-level–based) strategies, likely including some and unlikely all of those presented here. Teams are likely to have to balance the adoption of these strategies with the likelihood of adherence/compliance and player preferences and determine which of the strategies are likely to provide the most successful outcomes.

Consequently, whilst the recommendations provided in this article are targeted at the team level, there is an understanding that ubiquitous adoption is near impossible in real-world football and 2026 FWC reality. Therefore, the aim of this partner review is to provide an exhaustive framework, whilst acknowledging the aforementioned caveats, that helps teams to integrate their selective recommendations from the main review [1] into practice (where possible) with minimal disruption to football-specific preparations. The practice-compatible recommendations are presented in five sections:

1. ‘Travel and Early Arrival’ (Sect. 2).
2. ‘Heat Preparation and Mitigation’ (Sect. 3).
3. ‘Altitude Preparation’ (Sect. 4).
4. ‘Air Pollution and Seasonal Allergens’ (Sect. 5).
5. ‘Illness Prevention’ (Sect. 6).

**Fig. 1:**
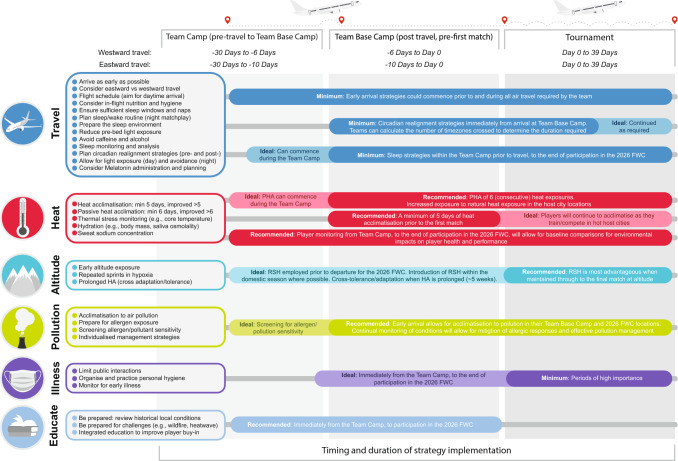
A visualised timeline for when teams could adopt the preparation and mitigation strategies for the environmental challenges at the Men’s 2026 FIFA World Cup. *FIFA* Fédération Internationale de Football Association, *FWC* FIFA World Cup, *HA* heat acclimation, *PHA* passive heat acclimation *RSH* repeated-sprint training in hypoxia

Finally, recommendations for when (e.g. temporally) the outlined guidelines could be adopted in practice are presented within each section and visualised in Fig. 1. The timing of adoption is organized into three approximate phases (with some inevitable overlap): (1) Team Camp (pre-travel to Team Base Camp); (2) Team Base Camp (post-travel and pre-first match) and (3) tournament.

## Travel and Early Arrival

The first challenge that players may face at the 2026 FWC is travel. Long-haul travel will be required for many teams to reach the 2026 FWC, whilst some players will need to travel from their domestic club locations to their home countries, which may also involve long-haul travel (e.g. Europe to South America). FIFA mandates arrival at least 5 days before a team’s first game at the FWC. However, physiologically and psychologically, teams will likely benefit from arriving as early as possible at their Team Base Camps or locations in the same/similar time zones where friendly matches can be played. In some cases, where logistics and schedules allow, players’ well-being may benefit from time spent in their home countries. Long-haul travel to the 2026 FWC will cause circadian misalignment (i.e. jet-lag), desynchronising physiological/psychological processes (e.g. mood, sleep) from the new destination time zone [46]. This misalignment will remain until the circadian system re-entrains to the new time zone [34, 47] and may result in negative consequences for health [e.g. gastrointestinal (GI) issues, mental health concerns] and performance (e.g. impaired cognitive and physical function) [32–35]. Thus, teams crossing multiple time zones require sufficient time to realign their circadian systems to the new time zone.

Teams can integrate the following evidence-based guidelines into practice with minimal disruption to their football-specific routines/schedules:

*Early arrival at the 2026 FWC:* All teams would benefit from arriving as early as possible at their Team Base Camp to support circadian realignment and allow players to acclimatise to the local conditions (i.e. heat, altitude, air pollution). For eastward travel, natural alignment to a new time zone takes 1 day per time zone crossed, and it takes half a day per time zone when traveling west [32, 48]. In worst-case scenarios, eastward-travelling teams (e.g. from Asia and Australia) will require a flight time of at least 13 h (longer journey times with stopovers may be required) and cross at least ten time zones. Westward travel (e.g. from the Middle East) may involve flights of up to 16 h (again, longer journeys with stopovers may be required) crossing up to ten times zones. Thus, arrival 10 days before the 2026 FWC is prudent for those travelling long distances eastward, and > 6 days would be recommended for those travelling westward.

A further benefit from arriving early to Team Base Camps is the chance for players to acclimatise to their new local environment. Humans are capable of naturally acclimatising to heat (Sect. 3), altitude (Sect. 4) and air pollution (Sect. 5). Specific acclimatisation strategies are outlined in the respective environmental challenge sections.

*Travel-specific strategies:* Whilst some teams may charter flights to the 2026 FWC, even those travelling commercially can opt for flight times and attempt to organise in-flight schedules to support circadian realignment:

1. *Flight schedule:* Teams can arrange flights to align with daytime hours at the Team Base Camp. This will allow meals to be scheduled to the destination time zone, which can aid in accelerating circadian realignment [32, 49]. A ‘sleep window’ between 13:00 and 16:00 at the destination time zone (optimal for napping [50]) is recommended to maximise rest/sleep during the flight. Eye masks, ear plugs/noise-cancelling headphones, maximum seat recline and limiting screen time can aid in-flight sleep [32]. For long-haul flights, sleep and rest are the priority [32, 38].
2. *Nutrition:* Where possible it is advisable for players to follow an individualised diet and hydration plan for the flight to meet individual energy requirements, prioritising the availability of fibre-rich foods, and avoiding high-calorie snack options [38]. Teams could consider contacting their airline well in advance of their flight to arrange this provision. It is recommended that players avoid alcoholic and caffeinated beverages because they can impair in-flight ability to fall asleep and the quality of any sleep [32].
3. *Hygiene:* Teams should prioritise in-flight hygiene (personal and food hygiene) and can determine the level of caution they wish to take [32, 38]. Players can be informed to avoid touching areas that carry high risk of micro-organisms that may spread illness (e.g. chair headrests, surfaces with high traffic of people), use hand sanitiser to disinfect high-touch areas around their seat (e.g. seat buttons, remote control, tray table) and wear a face mask (reliant on player willingness) to reduce the risk of virus propagation. However, teams may determine that regular and thorough hand-washing/sanitising is sufficient. In-flight food should be low risk for contamination to limit potential GI issues [32].

**Timing of strategy implementation:** Prior to and during all air travel required by the team.

**Duration of strategy implementation:** Players can adopt these strategies on all flight travel related to the 2026 FWC, including individual travel to join their national team, and all subsequent 2026 FWC travel. Players would benefit from being informed of these strategies prior to the team camp.

*Circadian realignment strategies:* Several pre- and post-travel strategies can accelerate circadian realignment to the destination time zone:

1. *Pre-travel alignment and sleep banking:* Players can be advised to shift their sleep/wake schedules by up to 2 h toward the destination time zone for 3 days before travel [51]; however, this is often difficult to implement due to personal and/or family commitments. Players may also extend their sleep duration to bank sleep prior to a period of sleep restriction (i.e. travel) [32]. This has been shown to reduce cognitive performance deficits during periods of sleep restriction and to accelerate performance recovery once normal sleep is resumed [52].
2. *Bright light exposure (day):* Morning light exposure is one of the most efficient ways to aid circadian realignment for those who have travelled eastward [32, 38]. For westward travel, afternoon and early evening light exposure is recommended [32, 38]. Teams could organise outdoor activities [e.g. pre- and/or post-breakfast (eastward) or dinner (westward) team walk or training] that expose players to natural light [53].
3. *Bright light avoidance (night):* Nighttime bright light avoidance helps prevent melatonin suppression and cognitive arousal, thereby aiding sleep preparedness. Specifically, players’ common areas and rooms could be dimly lit during darkness hours, and ideally, TV/device screen time should be limited (ideally none) at least 60 min before bedtime.
4. *Caffeine intake:* Caffeine may be used to increase wakefulness and reduce daytime sleepiness following travel but should ideally be avoided close to bedtime to avoid sleep disturbance [54, 55]. A ‘food first’ approach is typically preferred [56], and coffee is a suitable natural source of caffeine; however, the caffeine content in coffee varies and may be difficult to determine [56]. If teams aim to control caffeine intake more accurately, it can be taken as a supplement (tablets/chewing gum) but should be sourced from informed-sport (or similar) and batch-tested products to avoid contamination and inadvertent doping violations [57].
5. *Melatonin:* Bi-phasic (i.e. combined instant and slow-release composition) formulations can aid the phase shift towards the new destination and help keep players asleep during the night [58, 59], although, the efficacy can vary between individuals [60]. Melatonin administration is typically timed 1–2 h before bed when travelling east (i.e. advancing circadian rhythm) and early morning when travelling west (i.e. delaying circadian rhythm) [32]. The dose should be determined by the team physician [58, 59] and sourced from a reputable pharmaceutical supplier to avoid contamination and doping violations [57].
6. *Timing of exercise:* Initial exercise-based team sessions are often scheduled at the same time as light exposure – in the morning for teams travelling eastward and in the afternoon/early evening for teams travelling westward, as this timing facilitates accelerated circadian realignment [32, 36, 38]. Once players stop exhibiting symptoms of jet-lag/travel fatigue, these sessions can be planned according to team preferences. It is recommended that initial exercise sessions (first few days) are kept at low intensity and progressively advance to higher-intensity and skill-specific sessions [53].

**Timing of strategy implementation:** Immediately upon arrival at the Team Base Camp.

**Duration of strategy implementation:** Team travel requirement specific. Teams can calculate the direction of travel and number of time zones crossed to determine duration of these strategies (half a day per time zone crossed when travelling west; 1 day per time zone crossed travelling east [32, 48]).

The circadian realignment strategies are, in part, aimed at aligning players' sleep/wake cycles with the new destination time zone. Enabling players to get sufficient sleep will be important for maintaining their health and performance throughout the 2026 FWC [61–64]. Sufficient sleep is protective against the risk of illness [65] and supports recovery from the burden of travel [37] and training/match demands [61]. Elite athletes often suffer from disrupted and/or insufficient sleep [66, 67]. Late evening and/or night matches will be a feature of the 2026 FWC, reducing total sleep duration by ~ 3 h [62, 63] and disrupting players' bedtime routines and normal sleep/wake cycle [63]. During these late evening/night matches at the 2026 FWC, players will be exposed to factors that may impair sleep, including (1) bright stadium lighting [63, 68]; (2) activity that requires high arousal and focus [63]; (3) high-intensity exercise [69] that raises body temperature and delays the circadian cooling needed for sleep [70]; (4) consumption of caffeinated products [63, 68]; and (5) irregular sleep/wake times [71–73]. Indeed, elite football players self-report poorer sleep after night matches (kick-off after 6 pm) compared to training or daytime matches (kick-off before 6 pm) [68, 73]. This is supported by objective sleep data (actigraphy) showing that players spent ~ 1 h 40 min less time in bed and total sleep time was reduced by 1 h 32 min on match nights, compared to training days [68]. Additionally, travel fatigue may accumulate across the tournament as teams may be required to cover vast distances between venues. Although typically a temporary state that is resolved with one night of good sleep, accumulated travel fatigue from multiple flights and poor sleep quality can lead to performance decrements [74].

Adequate rest and sleep will provide support to players’ mental and physical recovery and help maintain performance throughout the tournament [75, 76]. Sleep hygiene strategies are evidence-based approaches to improving sleep quantity and quality in elite athletes with high ecological validity to football [63, 71]. However, their adoption depends on match, training and rest day scheduling:

1. *Sufficient sleep window:* Providing players with a sleep window (~ 10 h) is advised to allow players to get sufficient sleep. Elite athletes commonly report insufficient and/or poor-quality sleep [67], often averaging less than 7 h per night [64, 77, 78]. Sleep extension studies have shown that when athletes are provided with up to 10 h in bed, sleep duration is increased by up to 113 min [79–82], resulting in improvements in physical and cognitive performance [78].
2. *Sleep/wake routine:* Establishing and maintaining a consistent sleep/wake routine throughout the 2026 FWC will provide players with a stable circadian rhythm phase alignment that improves the propensity to sleep [63] (i.e. ease/readiness to go from wake to sleep and the ability to stay asleep [83]), an effective strategy for those suffering from insomnia [84]. Given that teams will play evening/night matches, a sleep window between 11 pm and 9 am can help limit the disruption to established sleep/wake timings. Although delayed sleep onset is likely inevitable after night matches, teams can aim to maintain consistent wake times, even if sleep is reduced. This helps avoid disrupting subsequent night sleep/wake routines [63], as shifts in sleep/wake times of more than 1 h can elicit a form of ‘social jet lag’ [85]. Total sleep duration on these days can be maintained via strategic naps.
3. *Naps:* Napping is an effective strategy to offset inadequate sleep [86] but should be used strategically to avoid impacting nighttime sleep. Naps are typically 20–90 min and taken between 13:00 and 16:00 [50]. Upon waking, allowing players at least 30 min for sleep inertia (i.e. the transition period between sleep and wakefulness with decremented cognitive function and alertness [87]) to dissipate [50] is beneficial. Teams could employ two types of nap:
  - *Prophylactic nap:* Increasing the total sleep duration in anticipation of known nights of disrupted or time-restricted sleep [50].
  - *Replacement nap:* Increasing the duration of a player’s total sleep following a night of disrupted or time-restricted sleep that has already occurred [50].
4. *Rest day sleep:* Teams can maintain consistent sleep/wake timings on rest days to avoid disruption to the sleep/wake cycle. Excessive time in bed is associated with fragmented and shallow sleep, which can impact subsequent nights of sleep [88]. Restricting the time available for sleep to specific hours improves total sleep time and efficiency (i.e. the ratio of time in bed vs time spent asleep) [63, 88].
5. *Rest day activity:* On rest days, it is advisable for players to remain lightly active [89], and exposure to natural/bright light (especially in the morning) [90] can be encouraged to ensure players get enough wakefulness time to elicit the drive for sleep at night [63].
6. *Sleep environment:* A cool (~ 18 to 24 °C [91–93]), dark and quiet room is optimal [70].
7. *Reduce pre-bed bright light exposure:* Light strongly influences sleep. While light exposure during the day helps to induce the drive for sleep at night [90], bright light exposure during the evening from floodlights [63], electronic devices [94] and/or bright indoor lights can suppress melatonin excretion [95] and delay the natural sleep/wake cycle [63, 95]. Furthermore, light and/or phone/tablet use in bed can increase cognitive stress and/or arousal prior to sleep and reduce sleep duration and quality [63, 96]. Thus, it is recommended to limit or eliminate the use of electronic devices and exposure to bright light before bed to optimise sleep for players [62, 63]. Player common areas and rooms can also be dimly light during the hours of darkness.
8. *Caffeine and alcohol:* Small doses (~ 100 mg) of caffeine intake 8 h before bedtime typically do not disrupt sleep [54, 55]; however, in a recent randomised clinical crossover trial, a dose of 400 mg taken 12 h before bed disrupted sleep by delaying sleep onset, altering sleep architecture and increasing sleep fragmentation [55]. Teams may wish to educate their players about caffeine intake and its impact on sleep to help maintain the established sleep/wake schedule. Similarly, alcohol consumption reduces sleep quality [97] and should be avoided entirely.
9. *Sleep monitoring:* There are a plethora of commercially available wearable devices that report to accurately measure sleep. However, they typically overestimate total sleep time and efficiency and underestimate awake time after sleep onset [98, 99]. Research-grade wearable activity monitors, however, have been shown to have good agreement with polysomnography (gold standard for sleep assessment [98]) when high-sensitivity (wake = > 80 activity counts) thresholds are set [100]. Importantly, subjective self-reported indices from players should not be ignored in favour of objective data [101–103].

**Timing of strategy implementation:** These strategies can begin during the Team Camp prior to travel to the 2026 FWC. Team schedules will dictate when rest day strategies [(4) and (5)] are implemented. Naps (3) can be employed ad hoc; if sleep monitoring is employed (9), teams can objectively prescribe naps for players who experience disrupted, limited and/or poor-quality sleep. Before and/or after night matches, teams may wish to bank or replace sleep that will be/was lost.

**Duration of strategy implementation:** From the Team Camp to the end of participation in the 2026 FWC.

Teams may wish to adopt a specific sleep strategy for night matches. Post-night match sleep can be improved with an acute sleep hygiene strategy, albeit most supporting data comes from highly trained amateur football players [71]. However, it involves a strict routine that may not suit all teams/player cultures. In a tournament-like scenario with matches at a stadium near the players’ accommodation, a study compared an acute sleep hygiene protocol to a no sleep hygiene protocol after two experimental night matches. The sleep hygiene protocol included (1) getting players into their bedrooms as early as possible post-match with dim lights, (2) cool temperature rooms (~ 17 °C), (3) no technology or light-based stimulation up to 30 min before bed and (4) lights were turned off at midnight [71]. The no sleep hygiene protocol involved allowing players to go about their normal activity until 02:00 am, when they were allowed to go to bed (i.e. a typical post-night match bedtime [72, 104]). The acute sleep hygiene protocol increased sleep duration by ~ 90 min compared to the no sleep hygiene protocol. However, there were no improvements in recovery markers or next-day training performance, perhaps due to more fragmented (increased wake episodes) sleep [71]. Over the duration of a tournament, even modest increases in sleep duration following night matches may have cumulative benefits, potentially reducing the negative impact of repeated night-time sleep restriction. However, this is speculative without specific data to confirm this.

## Heat Preparation and Mitigation

To prepare for and mitigate against the impact of extreme heat at the 2026 FWC, teams are unlikely to have the time and/or capacity to undertake gold standard approaches to HA (i.e. 2 weeks of daily 60–90 min exercise-based heat exposure [105–112]). European domestic/continental club competitions (where 70% of players played prior to the 2022 FWC [41]) finish as late as 11 days before the start of the tournament (UEFA Champions League Final: 30th May 2026). Therefore, innovative and time-efficient strategies may be required to induce the necessary physiological adaptations that protect athlete health and performance, whilst player monitoring technology can be used to assess the thermal stress on players objectively:

1. *Acclimatisation:* Teams whose Team Base Camps are in hot locations will have the opportunity to procure heat acclimatisation adaptations in their players. At least 5-days of living and training in the heat can induce some physiological adaptations that improve health and performance in the heat [113, 114]. The further a team progresses through the 2026 FWC, the greater the acclimatisation adaptations the players will accrue. The hottest time of day would provide the greatest heat stimulus; however, teams may avoid training during these times to ensure the quality of football-specific training remains high. The magnitude of adaptation to heat could therefore be limited, and teams may benefit from adopting additional HA strategies to acquire a fully heat-acclimated phenotype. Indeed, combined exercise and passive HA protocols have been effective in procuring a fully heat-acclimated phenotype in elite athletes [115, 116].
2. *Passive heat acclimation (PHA):* PHA offers a logistically feasible and practical method to induce heat adaptations without compromising football-specific training quality or schedules [117]. It involves daily or alternate-day passive heat exposure, with the most effective strategies including 30 min of post-exercise 40 °C hot-water immersion (HWI) [108, 118] or 80 °C sauna (with/without pre-exercise [119, 120]). A 60-min HWI exposure is recommended for PHA if used as a standalone strategy (i.e. no exercise) [121–123]. With well-coordinated communication between clubs and national teams, players may be able to integrate these strategies during their domestic club season. However, adopting a PHA strategy will require sufficient planning:
  - *Number of exposures:* Teams can aim for a minimum of six PHA exposures [108, 118]. Post-training PHA for at least 6 consecutive days offers the greatest efficacy to induce heat acclimation adaptations [108, 118]. If teams are able to follow the advice from Sect. 2 and arrive as early as possible, a PHA protocol can be completed before matches begin, complementing team training and limiting the impact of increased physiological stress on training and matches. PHA can be adopted without prior exercise and/or on alternate days; however, the time to induce heat acclimation adaptations will be increased and the magnitude of adaptations may be reduced [124].
  - *Recovery:* The additional physiological stress from a PHA protocol will necessitate the provision of time for adequate rest. Hydration status can be closely monitored to ensure fluid lost during training and PHA is replaced; this will be essential to protect players from EHI [125] and to ensure complete physiological adaptations. Players will likely require an increase in carbohydrate (CHO) intake given the combination of exercise and heat stress increases CHO utilisation [126].
  - *Supervision:* Players may not tolerate the full PHA heat exposures outlined above and may need to be gradually introduced to them. During PHA, players should be supervised at all times and should have constant access to fluids [108, 118] to avoid adverse EHI symptomatology [117]. Ideally, core temperature (Tc) would be monitored to assess individual heat stress objectively [117].
3. *Thermal stress:* Tc can be monitored non-invasively using ingestible telemetric capsules that record GI temperature, a technology that has been used regularly within elite [127–129] and recreational [130, 131] sport. To ensure accurate Tc data, capsules should be ingested at least 4 h prior to exercise [132]. Once activated via a data-logging device, the capsule continuously records temperature at set intervals (e.g. 5 or 30 s up to 5 min). When within ~ 1-m of the device, it will connect and display the latest temperature reading and download the data recorded on the capsule to the data-logging device. However, this technology imposes a high financial burden and may not be available to teams with limited budgets. Rectal temperature (Trec) is an accurate measure of Tc [133] and is relatively low cost, as probes can be reused with appropriate sterilisation. Yet, their use is invasive and particularly challenging outside laboratory settings and within football environments. These challenges have led, at least in part, to the development of several sensors that attempt to estimate Tc without direct measurement, modelling other physiological variables such as skin temperature and heart rate. These sensors cannot be used to accurately assess Tc responses to exercise and are known to over- and/or underestimate Tc by ≥ ± 0.3 °C (± 0.2 °C represents a physiologically meaningful change in Tc [134, 135]) compared to validated Tc measures [136–139]. Acutely assessing Tc provides teams with the ability to assess individual players thermal load before, during and after training/matches and adapt their heat mitigation strategies (e.g. cooling strategies) to reduce the risk of EHI/EHS and improve performance [109, 140]. Additionally, cumulative heat load over multiple days is a risk factor for EHI/EHS alongside a myriad of other individual factors (e.g. illness, dehydration) [141], and sufficient recovery is necessary to maintain player health and performance. Athlete and practitioner and/or medical staff knowledge of heat-based best practice can be limited [40]; if team practitioner/medical staff have limited experience with heat-based data, teams could/should seek external expertise.
4. *Cooling:* Cooling strategies before and during competition improve performance and reduce heat strain during exercise in the heat [42, 43], with some recent football-specific data demonstrating their efficacy [142]. Cold-water immersion (CWI) is considered the optimal cooling strategy [143] although its use is often limited by logistical constraints [1, 42]. Mixed-method cooling strategies are highly effective [42, 43], and can be applied to the body surface (e.g. external cooling: cold packs, ice-cold towels) or via ingestion (e.g. internal cooling: cold water, ice slushie).
  - *Pre-cooling:* Before training and matches, players can adopt a variety of cooling strategies. Tc was 0.7 °C lower post-warm-up in elite male/female Rugby Sevens players when wearing an ice vest (compared to no ice vest), with no impairment in warm-up physical performance characteristics (GPS-based data or counter movement jump metrics) and no discomfort/restrictions reported by players [44, 45]. Outside the warm-up, players can apply ice packs to large muscle groups, use ice-cold towels across a large body surface area and ingest cold/ice-cold fluids. Similar approaches have been effective in reducing physiological and perceptual thermal strain and improving physical performance in simulated football performance [144].
  - *Hydration/cooling breaks and half-time:* FIFA hydration/cooling breaks are typically implemented when WBGT reaches 32 °C (see [1] for an overview and critique of FIFA WBGT thresholds and cooling breaks); however, mandatory 3-min hydration breaks have been introduced throughout the 2026 FWC. In-line with FIFA policy, ice-water–soaked towels, cold water and cool boxes will be supplied [145]. Laboratory-based studies [145, 146] and one field-based study [142] demonstrate that cooling breaks can reduce thermal strain (lower body temperatures) and improve physical performance (improved running characteristics). Teams can aim to maximise the cooling time by applying cold towels as soon as possible and for as long as possible during these cooling breaks. The use of shade (e.g. large umbrellas) and educating players to seek shaded areas where available is also advisable [1]. During half-time, practitioners can attempt to maintain the changing room at a cool/cold temperature and maximise contact time with players to implement mixed-method cooling strategies, whilst minimising interference with player downtime and coach-led interaction [147, 148].
  - *Cooling strategy caution:* All cooling strategies should be trialled well in advance of competition and individualised where possible. Ingestion of cold/ice-cold fluids can cause GI distress, and aggressive internal cooling can impact the sweat response (i.e. delay sweating and evaporative heat loss) [149, 150]. Shivering should be avoided [109], and over-cooling of muscle temperature can negatively impact high-speed running performance [42, 109].
  - *Post-cooling:* Post-match cooling can serve a dual purpose, alleviating acute thermal strain and facilitating recovery during congested training and fixture schedules of tournament football. Where feasible, CWI is advisable, and the optimal strategy is 11–15 min in 11–15 °C water [151].
5. *Hydration status:* Dehydration is a key risk factor for EHI/EHS and decrements in performance during exercise in the heat [6, 141]. There are several methods that teams can consider employing to monitor hydration status (the methods used will be dependent on team resources/budget and other practice-facing constraints):
  - *Urine colour:* Players can self-report urine colour using a validated 6-point scale (dark = dehydrated, light/clear = hydrated) [152]. However, this method can only detect the presence, not extent, of dehydration and is invalid if large quantities of fluid have been consumed prior to urination [153]. Urine osmolality and/or specific gravity provide an assessment of hydration/dehydration, but obtaining multiple daily urine samples is logistically challenging. Finally, many factors can confound urine-based hydration assessments (e.g. time since last bladder void, ingested fluid composition and colour) [154].
  - *Body mass changes:* Daily standardised body mass measurements (e.g. nude body mass, same time of day, empty bladder [110]) and pre- to post-exercise measurements are valid and non-invasive measures to assess hydration (i.e. acute weight loss is typically loss of body water [153]). Any changes in body mass after training/matches should aim to be restored by the next day exercise-based activity [110, 153] (i.e. 1 g body mass loss = 1 ml fluid [154]). Body mass changes pre- to post-exercise of 2–2.5% are a threshold for concern regarding health and performance [155].
  - *Weight, urine colour, thirst (WUT) framework:* Combining daily body mass changes with the colour of first urine void and thirst perception upon waking under the WUT framework [156] provides an effective strategy for monitoring hydration status [157–159], with body mass change carrying the greatest weighting for estimating hydration status [157]. Table 1 outlines the WUT framework, including measurement, results and interpretation.
  - *Point-of-care saliva osmolality testing:* Hydration status can be rapidly determined with a point-of-care device that measures saliva osmolality. A testing strip is placed on the tongue, collects a saliva sample and is then read by the device [160]. Because saliva osmolality is highly variable between individuals, each player should first complete a euhydration protocol to individualise the results [161]. For optimal sampling, players should avoid fluid ingestion 10 min prior, swallow existing saliva and produce a fresh sample [160]. The result will then show whether the individual is hydrated or mildly, moderately or severely dehydrated. This device can be used to assess acute (pre- and post-training/match) and chronic (daily changes) hydration status and, alongside body mass changes, determine an individualised fluid replacement strategy. However, it does suffer similar limitations to urine colour, given recent food/fluid intake may confound results, and it does not determine the necessary fluid consumption required to rehydrate if a dehydrated state is detected.
6. *Sweat sodium concentration:* Players training/competing in the heat at the 2026 FWC will experience whole-body sweat losses and excretion of electrolytes [162], which are highly individual and influenced by HA status [163], sodium intake [164] and acute exercise intensity [165]. Sweat sodium concentration decreases linearly during HA, with reductions seen as early as 2 days of a HA protocol [166]. Whether teams adopt the outlined HA guidelines above [(1) and (2) in this section] or players arrive already acclimatised because they are based in hot locations, sweat sodium concentration is likely to change across the duration of the tournament. Baseline (i.e. pre-heat exposure) and periodic testing of sweat sodium concentration throughout the 2026 FWC will allow teams to adapt and optimise hydration strategies as players' acclimation status changes, and avoid hyponatraemia/hypernatraemia, although this has not previously been reported in football players. Sweat sodium concentration can be reliably tested using a field-based testing device and sweat patches placed on players skin prior to an exercise session in the heat. After the session, the patches are removed; the collected sweat is then squeezed into a tube, and the testing strips are used to measure the sodium concentration [167]. Within a real-world setting such as the 2026 FWC, it can be difficult to avoid sample contamination, and data from this device should be interpreted as a guide; a reliable and valid assessment of serum sodium concentrations and diagnosis of hyponatraemia will not be possible with this device.

**Table 1:** Overview of the WUT hydration framework

	Nude body mass	First void urine colour	Thirst
Result	(1) > 1% change from day before	(1) Dark	(1) Thirsty/very thirsty
	(2) < 1% change from day before	(2) Light	(2) Not thirsty
Interpretation			
1 × result (1)	Possible dehydration		
1 × result (2)	Possible euhydration		
2 × result (1)	Likely dehydrated		
2 × result (2)	Likely euhydrated		
3 × result (1)	Very likely dehydrated		
3 × result (2)	Very likely euhydrated		

*WUT* weight, urine colour, thirst

**Timing of strategy implementation:** PHA (2) could occur during the Team Camp prior to travel to the 2026 FWC, and perhaps with effective communication between national teams and players’ domestic clubs, it may be possible to begin PHA during the closing stages of the domestic club season. Acclimatisation (1) can begin as the players arrive at the Team Base Camp for teams located in hot environments. Player monitoring (3) to (5) can be implemented from the time that the team meet up prior to travel to identify baseline responses to objectively determine the influence of the tournament conditions on the players.

**Duration of strategy implementation:** Teams could aim for a minimum of 5 days heat acclimatisation (1) and six PHA exposures, ideally on consecutive days (2). Increasing the number of PHA sessions alongside natural heat exposure (in hot host city locations) will further increase the HA adaptations of the players. Furthermore, prolonging PHA exposures may be of benefit to teams who also have matches at altitude (full details in Sect. 4). Consequently, PHA could be implemented for the duration of the 2026 FWC. It will be important that teams provide players with appropriate nutritional support/guidance and are afforded adequate recovery/rest time. Finally, fixtures held in covered/indoor stadiums with air conditioning/climate control (Atlanta, Dallas, Houston and Vancouver) will reduce the burden of any heat and air quality–related challenges, with practitioners able to adopt their practice on a fixture-by-fixture basis.

## Altitude Preparation

The 2026 FWC will feature nine matches played at 1566 m and 2240 m, elevations that reduce aerobic capacity and prolong recovery between high-intensity efforts [17–19]. Teams from sea-level locations can implement strategies to mitigate against these altitude-related challenges. Traditional altitude acclimation strategies that drive haemoglobin mass expansion to improve oxygen transport and aerobic capacity [168] may not be practical in the short window before the FWC begins. Thus, innovative and time-efficient strategies are required:

1. *Acclimatisation:* For teams with matches at altitude (Mexico City, Guadalajara), data from the 2010 FWC suggests establishing the Team Base Camp at moderate altitude (950 m to 1700 m) may confer an advantage in matches played at altitude against those teams who stay at sea level [169], indicative of an acclimatisation effect. Thus, as per Sect. 2, teams would benefit from arriving as early as possible.
2. *Repeated-sprint training in hypoxia (RSH):* A typical RSH strategy involves two to three sessions per week, totalling eight to 12 sessions (~ 4 weeks), each with three to six repeated sets of four to eight maximal short-duration efforts ranging from 5 to 20 s with incomplete recovery [work-to-rest ratio (W:R) < 1:6] and 3–5 min active recovery between sets at a real or simulated altitude of ~ 3000 m [170]. This approach enhances neuromuscular efficiency and fatigue resistance [170, 171], enabling players to sustain repeated sub-sprint accelerations and improve match performance at altitude and sea level [172]. Shorter sprints (~ 6 s) with a 1:4 W:R appear to elicit neuromuscular adaptations, though the evidence comes from elite endurance cyclists rather than football or team sport athletes [173]. Access to artificial hypoxic facilities can be logistically challenging; however, early arrival at the 2026 FWC could reduce the required duration of simulated altitude. If well-planned, RSH could be well-integrated into team pre-travel and early arrival schedules. Teams should be cautious and balance any hypoxic stimulus with football-specific training and sufficient rest and recovery in players that may arrive at the FWC with accumulated fatigue from the domestic season [174].
3. *Prolonged HA:* Other than Mexico, who will play at least three games at altitude, teams may only play one match at altitude; however, if they progress through the stages of the FWC, they may face multiple matches in the heat. Prolonged HA (≥ 5 weeks [175]) can induce altitude acclimation-like adaptations in oxygen-carrying capacity [175] via cross-adaptation/tolerance [176]. Endurance performance improvements in hypoxia similar to those from altitude acclimation have been observed after just 10 days of active HA [177]. Thus, given the limited altitude exposure at the 2026 FWC, teams scheduled to play at altitude could derive more benefits from extending the PHA strategies outlined above to prepare for—and mitigate—the impact of altitude and heat.

Similar to heat-based practice, many football teams and their practitioners and medical staff may have limited experience and expertise with altitude/hypoxia exposures and may therefore benefit from external consultation from other fields (e.g. endurance disciplines) to ensure best practice is adopted and the players health and performance is protected.

**Timing of strategy implementation:** These strategies are best initiated during the pre-travel Team Camp for the 2026 FWC as they require implementation over several weeks. However, successful adoption depends on effective communication and collaboration with players’ domestic club teams. For RSH, access to hypoxic facilities will likely pose a barrier to implementation, but players may be able to begin these strategies during the closing stages of the domestic club season if player clubs agree.

**Duration of strategy implementation:** An RSH protocol would require up to 4 weeks but could be reduced if teams arrive at an altitude-based Team Base Camp. Prolonged HA can be adopted for as long as possible to maximise cross-tolerance/adaptation benefits.

## Air Pollution and Seasonal Allergens

Air pollution levels and seasonal allergens will vary between the host cities at the 2026 FWC. These have been outlined in detail in the main review [1], and teams can remain up to date with the local air pollution and allergen levels (where possible) so the most appropriate management strategies can be adopted:

1. *Acclimatisation to air pollution:* The greatest impact of air pollution on lung function and respiratory symptoms occurs within the first 1–2 days of exposure, but players with heightened sensitivity may experience persistent symptoms for several additional days [24–26]. Adaptation to air pollutants takes ~ 4 days [24–26], underscoring the importance of arriving early to the 2026 FWC and allowing time for acclimatisation to a new environment. Additionally, arriving at a match location with markedly different air quality (see [1] for a detailed overview of air quality data for each host city at the 2026 FWC) to the Team Base Camp 4 days early would also be advantageous to protect player health and performance, where schedules/logistics allow.
2. *Allergens:* Acclimatisation to allergens is not possible. Players with known allergies and/or those with high sensitivity to allergens can try to limit their exposure where possible and rely on individualised medication and treatment (e.g. non-sedating H_1_ antihistamines) [178]. Any medication-based strategy (i.e. timing and dosage) to mitigate the impact of allergens should be determined on an individual basis with the team physician.
3. *Screening:* Player symptomatology can be monitored to assess sensitivity to air pollutants and screened for allergies, where logistics allow, to guide appropriate and individualised management strategies [179, 180].

**Timing of strategy implementation:** Players could be screened (3) as early as possible to determine potential susceptibility to air pollution/allergens (e.g. during the season before the 2026 FWC). Early arrival, as per Sect. 2, will allow players to acclimatise to the environment at their Team Base Camp (i.e. air pollution). If conditions at match locations are markedly different from those at the Team Base Camp, teams can plan their arrival to allow for some acclimatisation where schedules allow. Allergy medications can be used when players are in locations with high allergen counts; this will require individualised management.

**Duration of strategy implementation:** Teams can continuously monitor local conditions at the Team Base Camp and match locations. Incidents such as wildfires can severely affect air pollution and aggravate allergies, which may impact the use of exposure-avoidance and medication management strategies.

## Illness Prevention

Mega-events such as a World Cup increase human activities that can exacerbate and/or facilitate the spread of airborne illnesses [181–183]. The effectiveness of illness countermeasures was demonstrated by the lowest overall incidence of illnesses in athletes ever recorded at an Olympic Games during the delayed 2020 Tokyo Olympics (i.e. 2021), which took place under extensive coronavirus disease 2019 (COVID-19) restrictions [184]. Whilst it is unrealistic to expect teams to replicate the stringent protocols used during the pandemic [185, 186], most players at the 2026 FWC will already be familiar with illness-prevention strategies. Measures that can be easily implemented within the Team Base Camp environment with minimal disruption to player and team schedules could be essential to minimising the impact of an illness outbreak (i.e. disruption to training/performance) [53, 187]:

1. *Limit public interaction:* The FWC will likely increase the spread of airborne illnesses, and close contact with the local population or spectators could be avoided where possible to reduce the risk of spreading an infection amongst a team [188, 189].
2. *Hygiene:* Personal hygiene will be important for players. Educating players on avoiding high-touch surfaces (e.g. door handles) and following strict hand-hygiene protocols can lower infection risk within a team [190, 191]. Providing ample, accessible and clearly signposted sanitising stations at the Team Base Camp is likely to improve hand-hygiene compliance [192] and aid limiting the outbreak and/or spread of illnesses [190].
3. *Early illness recognition:* Players can be encouraged to consult the team physician if they experience any abnormal responses to exercise and/or at the first sign of any illness symptomatology [190, 191]. Viral illnesses are most contagious during the first 3–4 days after symptom onset [190]. Prompt identification of symptomatic players will best limit the spread of an illness within the team [193]. Player isolation may be warranted when the risk of contagion is highest [190, 194], although, this will be illness dependent.

**Timing of strategy implementation:** These strategies can be implemented during the Team Camp prior to travel to the 2026 FWC; however, it should be noted that these measures place a high burden on players and are typically advised during periods of high importance (e.g. from the first 2026 FWC match to end of tournament participation). Thus, teams will need to balance the impact these types of strategies will have on players' well-being beyond illness and ensure players are well protected from infections. Team culture differences will likely dictate how strictly these strategies can/will be adopted.

**Duration of strategy implementation:** Similarly, the duration of adopting these strategies will depend on team culture. It is possible to employ these strategies during the Team Camp pre-travel period, upon arrival to the 2026 FWC and until the end of participation. It may also be prudent for players to stay informed of any illness outbreaks within their domestic club teams to reduce the risk of spreading infection upon arrival with the national squad.

## Environmental Awareness and Player Education

It will be important for teams to maintain ongoing awareness of the environmental conditions at both the Team Base Camp and across all their match locations. This enables the adoption of appropriate preparation and mitigation strategies and allows practice to be adapted to optimise player health and performance. While the main review provides a detailed overview of expected challenges at each host location [1], teams also need to remain alert to unpredictable environmental events that may deviate from historical norms. A heatwave could exacerbate heat stress in host cities already expected to be hot, while locations typically considered cool may experience extreme heat. In the event of a wildfire, air quality could be compromised in locations with typically good air quality, which combined with heat stress can be even more problematic [29, 30, 195]. Players may therefore exhibit allergen and poor air quality symptomatology in locations typically associated with good air quality. Access to real-time and localised information will be valuable to managing these situations. Teams can arrive at the 2026 FWC with well-planned preparation and mitigation strategies, while retaining flexibility to adapt to unexpected and/or exacerbated environmental challenges as they arise.

Educating players on the importance of the environmental preparation and mitigation strategies that teams plan to adopt will likely be key to increasing adherence. Educational interventions have been observed to increase the adoption of sleep hygiene protocols by elite athletes [70, 78] and evidence-based heat preparation [117, 128, 196], and improve nutrition knowledge [197]. Teams can actively integrate education into their 2026 FWC preparations and engage the players in learning about the environmental challenges they may face and the strategies they can adopt. Simply talking to players and/or disseminating educational materials has proven to be ineffective at increasing the adoption of evidence-based practice [198].

## Conclusion

The Men’s 2026 FWC will present players and teams with a unique combination of environmental challenges that have significant implications for health and performance. The specifics of these challenges have been outlined in the main review [1]. Extensive evidence-based preparation and mitigation guidelines to protect athlete health and optimise performance amid these environmental challenges are widely available. However, they often suffer logistical and practical barriers that preclude their use and are rarely underpinned by football-specific data, limiting their external/ecological validity. This partner review outlines how teams participating in the 2026 FWC can integrate evidence-based guidelines into their FWC-specific practices, with minimal disruption to football-specific training and match schedules, while best protecting player health and optimising performance during the tournament. Importantly, football-related needs may outweigh the adoption of the recommendations outlined here before and during the 2026 FWC. There may be individual player cases where strategies cannot be adopted and/or require an adapted and highly individualised approach (e.g. late arrival to the squad/tournament). Whilst teams can prepare and plan these strategies carefully, they also need to be ready to adapt to exacerbated (combined-stressor situations) and/or unexpected (compared to historical norms) environmental challenges across the host cities. Educating players on the performance and health-facing importance of these preparation and mitigation strategies is likely essential to encourage adoption and adherence. Finally, resource discrepancy between teams, rather than practice compatibility, may prohibit the use of one or more proposed mitigation strategies during preparation for, or competition at, the 2026 FWC.

## Declarations

### Data availability statement

No datasets were generated or analysed to produce this article.
